# Cortical Malformations: Lessons in Human Brain Development

**DOI:** 10.3389/fncel.2019.00576

**Published:** 2020-01-24

**Authors:** Lakshmi Subramanian, Maria Elisa Calcagnotto, Mercedes F. Paredes

**Affiliations:** ^1^Eli and Edythe Broad Center of Regeneration Medicine and Stem Cell Research, University of California, San Francisco, San Francisco, CA, United States; ^2^Neurophysiology and Neurochemistry of Neuronal Excitability and Synaptic Plasticity Laboratory, Department of Biochemistry, ICBS, Universidade Federal do Rio Grande do Sul, Porto Alegre, Brazil; ^3^Graduate Program in Biological Sciences: Biochemistry, Universidade Federal do Rio Grande do Sul, Porto Alegre, Brazil; ^4^Graduate Program in Neuroscience, Universidade Federal do Rio Grande do Sul, Porto Alegre, Brazil; ^5^Department of Neurology, University of California, San Francisco, San Francisco, CA, United States; ^6^Neuroscience Graduate Division, University of California, San Francisco, San Francisco, CA, United States

**Keywords:** human cortical development, MCD = malformation of cortical development, progenitors cells, neuronal migration, connectivity

## Abstract

Creating a functional cerebral cortex requires a series of complex and well-coordinated developmental steps. These steps have evolved across species with the emergence of cortical gyrification and coincided with more complex behaviors. The presence of diverse progenitor cells, a protracted timeline for neuronal migration and maturation, and diverse neuronal types are developmental features that have emerged in the gyrated cortex. These factors could explain how the human brain has expanded in size and complexity. However, their complex nature also renders new avenues of vulnerability by providing additional cell types that could contribute to disease and longer time windows that could impact the composition and organization of the cortical circuit. We aim to discuss the unique developmental steps observed in human corticogenesis and propose how disruption of these species-unique processes could lead to malformations of cortical development.

## Introduction

Malformations of cortical development (MCD) are an important and complex collection of neurodevelopmental disorders that underlie over 40% of medically refractory childhood seizures (Kuzniecky, 1994, 1995) with over three-quarters patients with MCD developing a seizure disorder (Leventer et al., 1999). A standout feature of MCD is their association with a broad range of cognitive deficits including mild to severe intellectual disability and autism (Guerrini and Dobyns, 2014). The heterogeneity in the genetic and phenotypic presentations that underlie MCD have limited our ability to classify these disorders and coincide with challenges to predict and manage these diseases. However, the increasing identification of genetic mutations have offered clues to common molecular pathways and cellular processes that are disrupted in cortical malformations. Mouse and rat models, the most frequently used to investigate the etiologies of MCD, have substantiated the clinical relevance of MCD-associated genetic mutations but have been unable to fully recapitulate the gross phenotypes observed in the clinical condition (Wong and Roper, 2016). A deeper understanding of human cortical development is necessary to more effectively apply the mechanistic findings from animal models to the disease state.

**FIGURE 1:**
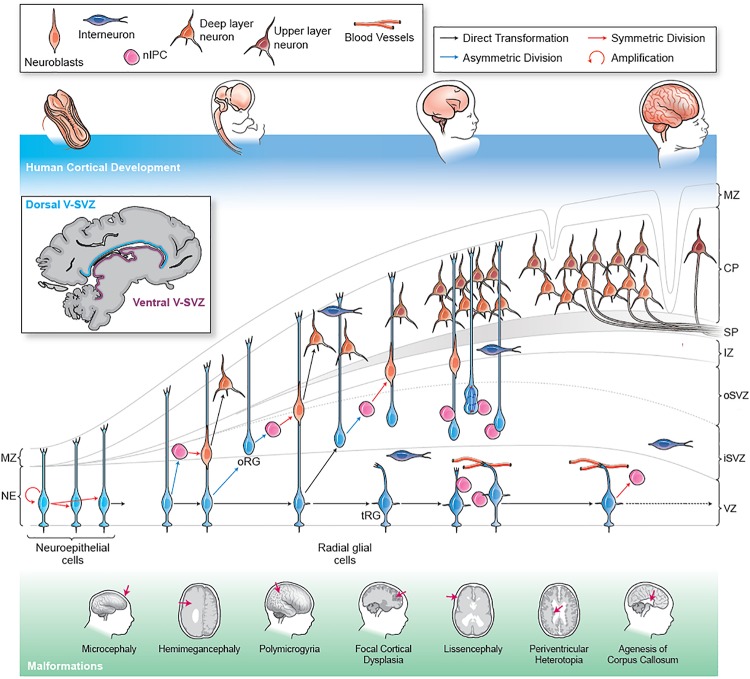
Human cortical development and stages of malformation. The human cerebral cortex forms early in the first trimester in the dorsal part of the telencephalon (forebrain). The human brain shows a rapid expansion in size and complexity during the 40 weeks of gestation as a result of extensive progenitor proliferation, migratory expansion and the generation of a complex connectivity pattern. During the first trimester, NE cells undergo symmetric division to expand the progenitor pool. NE cells elongate and convert into RG. By the end of the first trimester, RG are well established and can generate neurons (identified as migrating neuroblasts) directly through asymmetric division or indirectly by generation of IPCs. IPCs function as transient amplifying cells and can divide symmetrically one or more times to generate clones of neurons. Genetic mutations or environmental insults at this stage can cause microcephaly. In the second trimester, RG begin to give rise to RG-like cells that lack apical contact in the outer SVZ. These outer SVZ radial glia-like cells (oRG) are especially abundant in humans and other mammals with complex gyrencephalic cortices. oRG cells can generate neurons through IPCs and may contribute particularly to the generation of upper layer neurons. By the end of the second trimester, RG cells transform into truncated tRG. At this stage the RG scaffold is composed of the basal processes of the oRG cells. Proliferation errors or progenitor apoptosis in the second trimester can cause microcephaly or lissencephaly. Somatic mutations in mTOR pathway genes in NE, RG or oRG progenitors can result in FCD, HME or ME. Excitatory cortical pyramidal neurons are generated from RG and oRG progenitors via IPCs at the end of the first trimester. These neurons begin to migrate radially along the RG scaffold and until the middle of the third trimester. The pyramidal neurons maintain a radial organization as they migrate into and establish the cortical plate in an inside out manner, with the earliest generated neurons forming the deeper cortical layers while the youngest neurons contribute to the superficial layers. Errors in neuronal migration can result in heterotopias and lissencephaly. As they migrate, cortical pyramidal neurons begin to connect locally through transient connections in the subplate while they also begin to project axons that are myelinated by oligodendrocytes to form the cortical white matter. Errors in network connectivity can cause many forms of epilepsy, both *de novo* or secondary to other malformations along with ASD and schizophrenia. Errors of axonal projection lead to large scale connectivity defects like agenesis of corpus callosum. Toward the end of the second trimester, a combination of increased progenitor and neuronal numbers and rapidly expanding neuronal networks begins to generate physical stresses that contribute to the appearance of the main gyri. Over the course of the third trimester the secondary and tertiary gyrification of the cortex is established. Failure of gyrification may occur at any developmental stage leading to a range of malformations such as lissencephaly, polymicrogyria or pachygyria. Inhibitory interneurons migrate from ventrally located ganglionic eminences and appear in the cortex early in the second trimester. They migrate tangentially in the cortex along the marginal zone or in the subplate and SVZ and then move radially along the RG scaffold to integrate into the cortical circuits. Human interneurons continue to migrate into the cortex for a prolonged period through birth and early infancy. Failures of interneuron development, such as abnormal migration, arborization or maturation, can cause disinhibition within the cortical circuits resulting in epilepsy and cognitive dysfunction. Malformations of Cortical Development (MCD) (shown schematically at the bottom) arise at different stages along development. MZ, marginal zone; CP, cortical plate; IZ, intermediate zone, oSVZ, outer subventricular zone; iSVZ, inner sub-ventricular zone; VZ, ventricular zone; NE, neuroepithelium; RG, radial glia.

With this perspective, MCD offer an opportunity to decipher normal cortical development in the human brain. Proper development and organization of the mammalian brain requires the precise regulation of progenitor proliferation, cell type specification, and migration coordinated with neuronal differentiation, migration and cortical organization (Kriegstein and Alvarez-Buylla, 2009). Errors, due to gene mutations or environmental changes, can arise anywhere in this carefully choreographed series of events and result in alterations to cortical organization and a pathological states that are characteristic of MCD, including altered brain size, aberrant neuronal organization or clustering, and abnormal gyrification. In this review, we explore the neuro-developmental sequence in the human brain (Figure 1) and discuss various MCD associated with each of these stages, highlighting areas where human neurodevelopment differs from processes observed in mouse and rat models.

## Developmental Stage 1: Progenitor Proliferation

The human cerebral cortex is a complex structure showing a remarkable increase in size when compared to other vertebrates. This increase can be attributed to an evolutionary increase in the numbers and types of progenitor cells that give rise to the various types of cortical neurons and glia. The human cerebral cortex displays a remarkable radial organization of its excitatory neurons that is a result of the carefully organized radial architecture established early in development (Rakic, 2009). Cortical excitatory neurons are generated from a parent population of neuro-epithelial (NE) cells that are the founder cells in the nervous system located in the ventricular zone (VZ). These NE cells are arranged in a pseudostratified epithelial organization with apical and basal contacts. Early on in development, NE cells proliferate symmetrically to generate more NE cells and expand the progenitor pool (Subramanian et al., 2017). This expansion of the NE progenitors has been hypothesized to be one of the key factors that contribute to an increased number of progenitor cells in the human brain (Rakic, 2009).

Around the beginning of neurogenesis, progenitor cells begin to show characteristic morphological, molecular and mitotic changes as NE cells transform into radial glial (RG) progenitors. Similar to NE cells, RG cells have contact with both the apical and basal surfaces, but their basal processes get progressively longer and form the radial scaffold that not only support the cortical architecture but also provide a framework for newly generated neurons to migrate along and establish the cortical plate, giving rise ultimately to the radial organization of the mature cortex. RG cells show a dramatic increase in the number of asymmetric divisions when compared to NE cells. These asymmetric divisions give rise to two different daughter cells, one of which is a self-renewed RG cell. The other daughter cell can be either a neuron, that migrates along the radial fiber of its sister cell to the cortical plate or more often a basal progenitor cell that no longer has apical contact with the ventricular surface. The basal progenitors are called intermediate progenitor cells (IPCs) and are predominantly located in the subventricular zone (SVZ). They undergo several rounds of proliferative divisions (Rakic, 2009) before generating differentiated neurons in a terminal division. At the end of neurogenesis, RG progenitors transform into translocating progenitor cells that lose contact with the apical surface and migrate through the cortex, eventually generating astrocytes. These translocating RG have been described extensively in multiple species including rat, ferrets, monkeys and humans (Schmechel and Rakic, 1979; Voigt, 1989; deAzevedo et al., 2003; Noctor et al., 2004).

A subtype of radial glia called the outer radial glial cells (oRGs) have been shown to generate neurons in humans, non-human primates and carnivores (Fietz et al., 2010; Hansen et al., 2010; Reillo et al., 2011; Kelava et al., 2012). Much smaller numbers of these cells have also been identified in mice (Shitamukai et al., 2011; Wang et al., 2011; Kalebic et al., 2019). Human oRG cells exhibit a characteristic mitotic behavior called “mitotic somatic translocation” (MST). The parent oRG cell moves rapidly along the basal process in the direction of the pial surface just prior to mitosis. This dramatic movement depends on the integrity of the basal process and contributes to the expansion of the oSVZ (Fietz et al., 2010; Ostrem et al., 2014; Kalebic et al., 2019). Recent studies have shown that the oRG cells become the predominant progenitor cell in the human cortex by mid-neurogenesis 17 gestational weeks (17 GW). At this stage, oRG cells also become the main contributor to the radial scaffold that supports the development of the cortical architecture as the vRG cells transform into truncated forms whose basal processes no longer reach the pial surface (Nowakowski et al., 2016). In humans, oRG cells are generated from ventricular radial glia (vRG) by a process that resembles epithelial-mesenchymal transitions (LaMonica et al., 2013; Pollen et al., 2015). vRG cells lose apical contact with the ventricular surface and translocate away into the SVZ to form an expanded progenitor rich, outer subventricular zone (oSVZ). Similar to vRG cells, oRG cells undergo multiple rounds of asymmetric division where they self-renew and generate daughter IPC cells.

Malformations of cortical development have been described that are associated with multiple progenitor cell types. In particular, changes to patterns of progenitor proliferation appear to be responsible for several developmental malformations. Progenitor proliferation consists of several events that are susceptible to errors leading to cortical malformations. These errors include but are not limited to changes in the proliferation rate, changes to symmetric or asymmetric division patterns, errors in mitosis resulting from changes to spindle orientation or centrosome maturation and distribution, errors in apical or basal attachment of progenitors affecting the position of the mitotic progenitors and increased progenitor apoptosis (Guarnieri et al., 2018; Pinson et al., 2019). Several of these errors may be the result of germline or somatic mutations in the patient, but there may also be environmental causes including viral infections *in utero* that predominantly affect progenitor cells.

### Microcephaly

Primary microcephaly is a condition in which patients exhibit a marked decrease in the size of the head and the brain (>3 SDs over mean head size for the same age and gender). Although cortical organization is mostly preserved in the smaller brain, patients often have significant intellectual disability (Jayaraman et al., 2018). Microcephaly is predominantly associated with a decrease in progenitor numbers. This decrease can be due to decreased proliferation, changes in patterns of symmetric and asymmetric divisions and increased progenitor cell death.

Primary microcephaly is present at birth and can be caused by both genetic mutations and environmental insults like infections and toxins. Viral or parasitic infections such as Cytomegalovirus, Influenza, Herpes Simplex and Zika virus as well as parasitic infections like Toxoplasma gondii have all been linked to primary microcephaly (Devakumar et al., 2018). The microcephaly associated with the recent Zika virus epidemics have highlighted the role of progenitor cell proliferation in determining the size of the cerebral cortex in humans. Mouse studies, human *in vitro* models and studies on the developing human brain described widespread infection and consequent cell cycle arrest and apoptosis in infected NE, vRG and oRG cells (Li et al., 2016; Onorati et al., 2016; Retallack et al., 2016). Environmental toxins like alcohol in fetal alcohol syndrome have been shown to cause microcephaly through diverse effects on the developing neural tissue. Toxins and their metabolic by-products affect the survival and viability of progenitor and immature cells (Ehrhart et al., 2019). In addition, toxins can alter normal developmental signaling pathways including growth factor signaling, cause cytoskeletal disorganization in developing progenitor cells, alter the epigenetic landscape of developing neural tissues, initiate abnormal inflammatory responses, and alter patterns of programed cell death (Petrelli et al., 2019).

Abnormal cell biological changes in neural progenitor cells appear to be closely linked to primary microcephaly. The organization, maturation and distribution of the centrosome, and thus the organization of the spindle fibers appears to be especially vulnerable to these errors. The first gene mutations identified in patients with microcephaly, such as MCPH1, ASPM, CDK5RAP2, and CENPJ (Bond et al., 2002, 2005; Jackson et al., 2002; Zhong et al., 2006), had critical roles in the centrosome maturation and spindle organization and the list has been growing continually (Jayaraman et al., 2018). Centrosomes regulate the mitotic spindle and control both the ability of the progenitors to divide as well as the proper distribution of chromosomes across the two daughter cells. Errors in spindle organization can result in abnormal chromosomal numbers following cell division resulting in apoptosis of the daughter cells. Abnormal centrosome localization can also lead to abnormal orientation of the mitotic spindle, leading to a premature shift from proliferative symmetric divisions of ventricular progenitors to asymmetric divisions. Such a shift rapidly depletes the progenitor pool resulting in a smaller brain size. Mutations in the ASPM gene account for up to 40% of all autosomal recessive microcephaly (Pirozzi et al., 2018). Recent studies using genome editing approaches to knock out ASPM expression in the gyrencephalic ferret cortex (Johnson et al., 2018) have highlighted the role of this centrosomal protein in regulating the transition of apical vRG cells into basally located oRG cells. Such a change in progenitor sub-type results in fewer proliferative divisions. Further, loss of ASPM appears to disrupt the apical polarity complex that anchors vRG cells to the apical surface, leading to the delamination of the apical progenitors and an increase in the number of oRG cells. It is likely, therefore, that the effects of ASPM on the apical to basal transition in progenitors may be independent of or in addition to the effects of ASPM on the mitotic apparatus.

Mutations in the WDR62 gene are also frequently identified in patients with microcephaly. Mutations in human WDR62, however, result in a wide range of cortical malformations including microcephaly, pachygyria (unusually thick gyri) as well as callosal defects, lissencephaly and schizencephaly (Bilgüvar et al., 2010; Yu et al., 2010). Although gene knockout (KO) studies in mice have been very useful in understanding the role of WDR62 protein at the spindle pole as well as its interactions with ASPM (Jayaraman et al., 2016), other cortical phenotypes are not replicated in mice, which could be due to its absence of cortical folding. A recent report using gene edited human cerebral organoids has suggested a role for WDR62 in regulating progenitor proliferation as a result of delayed disassembly of the primary cilium, leading to cell cycle arrest and progenitor cell death (Zhang et al., 2019).

Other mutations associated with microcephaly appear to be involved in DNA regulation including DNA repair and chromatin organization (Jayaraman et al., 2018). These mutations appear to either affect cytokinesis of mitotic progenitors as a result of abnormal chromatin organization or increase progenitor apoptosis as a result of abnormal chromosome numbers.

### mTORopathies and Progenitor Cells

There are also a spectrum of cortical malformations that are associated with abnormal cortical organization. These encompass a wide range of disorders involving local overgrowth or disorganization of specific cortical regions or cerebral hemispheres (Focal Cortical Dysplasias- FCD, Tuberous Sclerosis – TSC, partial or Hemimegalencephaly- HME) to enlargement of the entire cerebral cortex (Megalencephaly – ME) or the entire head (macrocephaly) (Pavone et al., 2017). While megalencephaly might contribute to an increase in head size, macrocephaly is more commonly associated with changes to the bone structure, vasculature or hydrocephalus. In contrast, FCD, TSC, HME and ME appear to be a series of related conditions resulting from specific changes to cortical architecture and size. In addition to abnormal cortical organization involving enlarged and aberrant neurons, disruptions of cortical layering, balloon cells as well as cortical tubers in TSC, patients with these conditions also suffer from intractable or medication-resistant epilepsy. In addition, some patients might also have some form of intellectual disability or autism spectrum disorders.

These malformations have been linked to mutations that hyper-activate the PI3K-AKT-mTOR signaling pathway. As a major signaling pathway regulating cell growth and proliferation, over-activation of the mTOR signaling pathway is thought to have an especially significant effect on progenitor cells, either their proliferation or their proper differentiation (Iffland and Crino, 2017). Sequencing studies from surgically resected patient lesions have identified mutations in multiple genes that are a part of or signal to the mTOR pathway AMPK, PI3K, AKT, PIK3CA, GATOR1 complex (DEPDC5-NPRL2-NPRL3), MTOR, TSC1, TSC2, PTEN, and STRAD (Iffland and Crino, 2017; Marsan and Baulac, 2018). While some of these mutations are germline mutations, multiple studies have demonstrated that in many cases the mTOR activating mutations are uniquely present within only a subset of cells associated with the lesion. This led to the hypothesis that the somatic mutation likely occurred in a single progenitor cell sometime in early neurodevelopment (Crino, 2011), with the more widespread malformations arising out of a mutagenic event in a progenitor cell at an earlier stage of development. Thus, the severity of the malformation may be directly linked to the stage of cortical development, with more severe malformations being the result of earlier mutations whereas mutations that occur later in development result in smaller malformations. Recent studies in mice have established that cortical malformations occur when the mTOR-activating mutation is present within the dorsal lineage (D’Gama et al., 2017). Studies using *in utero* electroporation approaches in rat and mouse models have been able to recapitulate the pathological and seizure phenotypes of mTOR-mediated cortical malformations by manipulating the molecular players in the mTOR signaling pathway. *In utero* electroporation approaches offer an elegant means of modeling the effects of somatic mutations arising in the fetal cortex in a focal subset of neural cells at varying developmental time-points. CRISPR-mediated gene deletion of DEPDC5 in rats (Hu et al., 2018) and activation of mTOR signaling in mice using constitutively active mTOR kinase or Rheb proteins (Lim et al., 2015; Nguyen et al., 2019) have been shown to cause abnormal neuronal morphology and migration defects in the developing cortex. Furthermore, the severity of the seizure phenotype appears to correlate well with the extent of the electroporation, providing evidence linking the severity of malformations with the timing of the mutagenic event.

Patients with mTOR-mediated cortical malformations also appear to have a combination of germline and somatic mutations within the mTOR pathway leading to the theory that the severity of the malformation may depend not just on the timing of the second somatic mutation but on a combination of the genes affected by the somatic mutation and the timing of the second hit (D’Gama et al., 2017; Ribierre et al., 2018). The two-hit hypothesis presents an intriguing model for human mTORopathies, particularly in light of recent evidence that mTOR signaling in human cortical progenitors is uniquely active only in the oRG cells (Nowakowski et al., 2017). oRG cells predominantly contribute to neurogenesis in humans between the middle and end of the second trimester (Hansen et al., 2010). This developmental time window may therefore be particularly vulnerable to the effects of mTOR-activating somatic mutations.

### Cortical Folding and Progenitor Cells

The increase in the number of progenitor cells by proliferative expansion has been suggested to be a contributor to the gyrification, cortical infolding, of the human cortex. The progenitor-driven model of cortical folding was initially proposed following the development of folded brains in mice with excessive progenitor cells following constitutive activation of beta-catenin signaling (Chenn and Walsh, 2003). The more recent discovery of the expanded population of oRG cells in many gyrencephalic species (Martínez-Cerdeño et al., 2012) has led to the hypothesis that the presence of this expanded progenitor pool might be an important driver of cortical folding (Stahl et al., 2013; Wang et al., 2016; Borrell, 2018; Llinares-Benadero and Borrell, 2019). A recent study of the developing macaque cortex has put forth the idea that gyrification is a result of the expansion of the oSVZ progenitors but is driven by gliogenesis rather than neurogenesis (Rash et al., 2019). Several cortical malformations have been associated with abnormal gyrification including smooth brain (lissencephaly), excessive gyrification (polymicrogyria), and increased gyral thickness (pachygyria) but it is unclear at this time what the role of progenitor cells are in the generation of these malformations. Key gene mutations associated with gyrification defects including LIS1 and FLNA appear to regulate mitosis and early differentiation of progenitor cells in mouse models, affecting the orientation of spindle fibers, cell cycle length and cytokinesis (Vallee and Tsai, 2006; Fallet-Bianco et al., 2014; Moon et al., 2014; Sun and Hevner, 2014; Lian et al., 2019). Studies on human cerebral organoids generated from patients with Miller-Dieker syndrome (MDS) identified specific changes in the mitosis of oRG cells (Bershteyn et al., 2017). It remains to be seen, however, how changes in progenitor cell proliferation and migration relate to aberrant cortical folding patterns associated with gyral malformations.

## Developmental Stage 2: Neuronal Migration

A fundamental property of the developing brain is that newborn neurons must leave their site of origin to migrate varying distances to their target regions. Within the cortex, they leave the V-SVZ and reach their appropriate location within the developing cortical plate (CP), the future six-layered cortex (Buchsbaum and Cappello, 2019). This process happens in a highly regulated pattern in the mammalian brain to correctly establish the distinct laminae of the cortex. The cortex is also one of the most complex parts of the brain across species not simply in size but in anatomical architecture and cellular organization. Errors in the movement and placement of incoming neurons, therefore, can have consequences in the final cortical network. These fall under the category of MCD and can manifest with a wide spectrum of phenotypes, including seizures and cognitive disability.

For excitatory/pyramidal cells, migration depends on the RG process of neural progenitors to serve as a scaffold between the progenitor niche and the CP (Noctor et al., 2001). Radial migration is the primary mode of excitatory neuron movement in the mammalian neocortex. Post-mitotic neurons leave the V-SVZ using locomotive behaviors to travel along the RG fiber to reach the CP. This longitudinal scaffold provided by vRG cells underlies the protomap or radial unit hypothesis for how the cerebral cortex is built (Rakic et al., 2009). The young neuronal progeny generated by positionally related progenitors are kept together by the physical restraints of the RG fibers. Thus the cortical surface can expand with individual neurons maintaining their spatial, and possibly molecular, identity within the developing cortical layers. Once arrived, they undergo somal translocation to position themselves within the correct lamina. Their migration is regulated by several key factors including gap junctions between RG and the migrating neuron (Elias and Kriegstein, 2008) and the guidance by the extracellular protein Reelin, expressed at the marginal zone by Cajal–Retzius cells (Hirota and Nakajima, 2017). These elements influence cytoskeletal dynamics and adhesion properties of the migratory neurons and a disruption, either genetic or environmental, leads to disorganized formation.

Inhibitory neurons (interneurons) are produced in the ventral/subpallial embryonic brain within the ganglionic eminences and undergo a long “tangential” migration to reach their appropriate layers in the CP (Marin et al., 2010). Their migration undergoes a more complex pattern characterized by saltatory motion where interneurons have abrupt changes in speeds and accentuated pauses (Bellion et al., 2005). These long-range movements observed by interneurons are guided by a variety of cues. Neuregulins influence ERBB4-expressing MGE-derived interneurons and CXCR4 and CXCR7 chemokine receptors mediate migration in response to stromal-cell-derived factor 1 (SDF1) present in the marginal zone and intermediate zones of the developing cortex (Tiveron and Cremer, 2008; Li et al., 2012). Interneurons eventually change to radial migration as they enter the CP. Dysregulation of these processes can lead to disorganized lamina and abnormal placement of neurons within the gray and white matter.

### Lissencephalies

Lissencephaly, or “smooth brain,” is a set of conditions where the surface involutions (sulci and gyri) of the brain are missing or abnormal due to defects in neuronal migration. One report has implicated infection by cytomegalovirus, but most of our understanding of lissencephaly comes from the identification of associated genes that involve different aspects of cellular movement, including cytoskeletal integrity and extracellular matrix (ECM) interactions (Joseph et al., 2008; Mitchell, 2015). The first genes identified in patients with cortical malformations highlighted the importance of the cytoskeletal machinery. LIS1 and DCX mutations were identified in patients with lissencephaly (Reiner et al., 1993; des Portes et al., 1998; Fox et al., 1998; Gleeson et al., 1998); both genes encode microtubule-associated proteins. DCX is an X-linked gene and mutations in men result in complete lissencephaly while in females, the mutation is associated with ectopic neuronal layering, such as in subcortical band heterotopia or double cortex (Pilz, 1998). The product of LIS1 gene regulates transport along the microtubule motor protein, dynein, and the DCX protein, doublecortin, regulates microtubule stability and signaling during migration (Faulkner et al., 2000; Tanaka et al., 2004). Mutations in one of the seven tubulin isoforms, the proteins that polymerize into microtubules, are found in a broad spectrum of malformations (Bahi-Buisson et al., 2014; Bahi-Buisson and Cavallin, 2016). Tubulin-related malformations, or tubulinopathies, demonstrate the high overlap between different MCD and the intimate relationship between progenitor cell divisions and neuronal migration in normal cortical development. Tubulin is fundamental to the function of microtubules and the centrosome, thus defects can impact on both progenitor proliferation and neuron migration. Many tubulin mutations are associated with microcephaly, highlighting the importance of microtubules on the mechanics of cell division (Chakraborti et al., 2016). However, tubulinopathy phenotypes also include heterotopic cortical layering and abnormal gyration including microlissencephaly, classic lissencephaly (agyria), subcortical band heterotopia, and polymicrogyria-like cortical dysplasias (Jaglin and Chelly, 2009; Chakraborti et al., 2016).

The ECM is another arena where disrupted interactions between neural progenitors, migrating neurons, and supporting external macromolecules can lead to abnormal cortical layers and loss of gyration as seen in MCDs. ECM is a complex lattice of macromolecules including collagens, proteoglycans, and glycoproteins that occupies the extracellular space in tissue (Maeda, 2015). It serves many functions including as an adhesive substrate for cells and a reservoir for signaling molecules such as chemokines. The glycoprotein Reelin is the classic and most studied member of this group (for more detailed reviews please see Ishii et al., 2016; Lee and D’Arcangelo, 2016). Reelin mutations have been associated with lissencephaly with cerebellar hypoplasia, and the focus has been on expression by Cajal–Retzius cells at the meninges and the early embryonic role in regulating excitatory neuron migration for proper cortical layering. However, Reelin expression and members of the Reelin signaling pathway persists postnatally in the human brain (Abraham and Meyer, 2003; Deguchi et al., 2003). The function of Reelin at the end of gestation and in the early postnatal period is unknown. Reelin localization outside of the ECM and along dendrites suggests a role in synaptic remodeling and neuronal maturation (Roberts et al., 2005; Stranahan et al., 2013). Yet, Reelin receptors have been shown to function in neuronal migration in the postnatal rostral migratory stream (Andrade et al., 2007) and mouse cortical interneurons born at the end of gestation continue to respond to Reelin signaling in the brain, even at ages where Cajal–Retzius cells have disappeared (Hammond et al., 2006). Thus, Reelin could function later in cortical development to regulate neuronal migration, especially in the human brain where neuronal migration continues into infancy (Sanai et al., 2011; Paredes et al., 2016).

Other ECM components have been implicated in cortical malformations. Dystroglycans complexes serve as a physical link between the cytoskeleton and the ECM; their function is greatly modified by post translational changes such as glycosylation (Barresi, 2006). While “dystroglycanopathies” classically manifest as congenital muscular dystrophy, mutations in glycosyltransferase enzymes such as POMT1 and LARGE have also been shown to present with brain malformations such as cobblestone lissencephaly and polymicrogyria (Kano et al., 2002; Balci et al., 2005; Meilleur et al., 2014). A rare cortical malformation, bilateral frontoparietal polymicrogyria (BFPP), arises from mutations in the adhesion G-protein coupled receptor GPR56 (Piao, 2004). Collagen III is the ECM ligand for GPR56. Postmortem human and mouse model studies have shown that loss of GPR56 function leads to abnormal ECM organization within the meninges, resulting in pial heterotopias at the cortical surface and cobblestone lissencephaly (Li et al., 2008; Singer et al., 2013). The signaling pathways that are affected by these ECM-associated mutations is not known.

### Heterotopias

Cortical malformations can also appear as neuronal clusterings, or heterotopias, in abnormal locations. These are commonly identified as periventricular heterotopias (PVH) arising from the ventricular wall or subcortical heterotopias within the cortical layers. Despite their focality, heterotopias have significant impact: the vast majority (over 80–90%) of patients with these localized collections have seizures (Srour et al., 2011; Barkovich et al., 2015). The phenotype becomes more severe if associated with more expansive heterotopia size or other types of cortical malformations (d’Orsi et al., 2004).

The most commonly identified gene in patients with PVH is FLNA (Fox et al., 1998; Sheen et al., 2005) that encodes for filamin A, an actin-binding cytoskeletal protein and serves as a scaffolding protein, binding as many as 45 different proteins (Lian and Sheen, 2015). Other cytoskeletal members linked to heterotopias in a genetic screen of individuals with PVH include TUBG1, KIF2A, and microtubule-associated protein 1B (MAP1B) (Poirier et al., 2013; Heinzen et al., 2018). FLNA mutant models in mice and rats have also shown defects in neural progenitor proliferation and abnormal RG scaffolding in addition to arrested neuronal migration from the V-SVZ (Nagano et al., 2004; Carabalona et al., 2012), highlighting the complex interplay of different developmental cell types in the brain. Further support of heterotopias involving more than neuronal migration was the association of PVH-microcephaly with mutations in the ARFGEF2 gene (Sheen et al., 2004). ADP-ribosylation factor guanine exchange factor 2 (ARFGEF2) directs vesicle trafficking and fusion and heterotopias from mutations in this gene are linked to a disrupted neuroependymal lining and abnormal cell-cell contact within the ventricular zone (Ferland et al., 2009). Progenitors and the neuroepithelium have also been highlighted in PVH by the presence of mutations in DCHS1 and FAT4, members of the protocadherin family; both these protocadherins are highly expressed in the ventricular zone of early fetal human brains compared to the intermediate zone and the developing cortical plate (Cappello et al., 2013).

Modeling these heterotopias in mouse and rat brains have highlighted differences between human and rodent brain development and suggest divergent regulatory processes at play in different species. Neither FLNA KO or the FAT4 KO mice develop heterotopias (Carabalona et al., 2012; Badouel et al., 2015). Furthermore, focal knockdown of either FAT4 or DCHS1 in embryonic mice led an increase in progenitor proliferation in addition to accumulation of cells in the mouse ventricular zone (Cappello et al., 2013). However, 3D modeling using human iPSC-derived organoids did replicate this hyperproliferation due to FAT4 or DCHS1 mutations (Klaus et al., 2019). Instead, these organoids revealed morphological and transcriptomic changes in mutant progenitor cells together with abnormal migratory behaviors including increased paused times. Heterogeneity was also observed in that despite all neurons bore the same mutation, only a subset had abnormal migration and formed clusters. Taken together, studies of heterotopias demonstrate the intimate link, both physical and molecular, between neural progenitors and migratory young neurons.

## Interneuron Development and Malformations

The specific role of the GABAergic interneuron in MCD pathology and whether interneurons are directly disrupted or are secondarily affected by the abnormal development is unknown. Changes in interneuron distribution and number in MCDs have been observed in both the lissencephaly and FCD human cortex (Pancoast et al., 2005; Medici et al., 2016; Nakagawa et al., 2017), with parvalbumin-expressing subtype was the most affected interneuron. In patients with MDS (lissencephaly involving 17p13 deletion), the number of calretinin-expressing interneurons were abnormal in the fetal cortex but were no different at postnatal childhood ages when compared to the number in “control” brains; this suggested a specific effect on migration (Pancoast et al., 2005). Analysis of surgical resections from patients with FCD have also tried to shed light into how interneurons contribute to the seizure phenotype. Surgical tissue resected from FCD patients had a reduction in the frequency of spontaneous inhibitory currents onto pyramidal cells compared to currents in control (non-FCD) resected tissue (Calcagnotto et al., 2005); the change in inhibition was associated with abnormal interneuron distribution and altered GABA reuptake kinetics. A defining feature in FCD type II is the presence of morphologically aberrant cells, including cytomegalic neurons and balloon cells (Najm et al., 2018). The origins of these abnormal cells is unknown, but electrophysiological studies of cytomegalic neurons show that they have membrane properties that could render them as a seizure-generating (Wuarin et al., 1990; Tasker et al., 1992, 1996; Cepeda et al., 2006). Interestingly, in cases of severe FCD, cytomegalic neurons were found to be interneurons with more complex arborization and, unlike pyramidal cytomegalic neurons, had hyperexcitable properties, including the presence of spontaneous depolarization (André et al., 2007).

While all these studies suggest a mechanism for epileptogenesis in FCD patients, it remains unknown whether the changes were secondary to the emergence of the dysplasia. In a toxin-induced gyrencephalic model for cortical dysplasia, the MAM (methylmethoxymethanol)-exposed ferret, interneuron migration is disorganized and is associated with a disorganized distribution of Calbindin- and Parvalbumin-expressing interneuron subtypes (Poluch et al., 2008). This was hypothesized to be a non-intrinsic, or indirect, phenomenon as transplanted interneuron precursors cells from the MAM-treated ferret brain migrated normally in the normal (non-MAM-treated) cortex. One gene that directly ties abnormal interneuron development to MCD is the ARX (aristaless related homoebox) gene. ARX mutations have been associated with diverse symptoms including agenesis of the corpus callosum (ACC) and lissencephaly; XLAG syndrome includes severe cases associated with abnormal genitalia (Bonneau et al., 2002; Kato et al., 2004). Glutamatergic neurons do not express ARX though changes in ARX expression can indirectly affect their radial migration (Friocourt et al., 2008). The lissencephalic ARX brain is a 2–3 layered cortex with diminished neuronal populations and loss of interneurons (Forman et al., 2005; Okazaki et al., 2008; Marcorelles et al., 2010). ARX is a transcription factor that acts at several developmental stages including interneuron progenitor proliferation and neuronal migration (Friocourt et al., 2008; Friocourt and Parnavelas, 2011), though many questions remain about its exact function. The generation (Arshad et al., 2016), migration, and maturation of human cortical interneurons take place over a long timeline (Nicholas et al., 2013; Paredes et al., 2016; Close et al., 2017); therefore there are several points along their developmental trajectory where interneurons may directly or indirectly become dysfunctional and lead to abnormal circuit formation and connectivity.

## Developmental Stage 3: Connectivity

As discussed, the telencephalon develops through a sequence of spatiotemporally coordinated events: cell proliferation, migration, differentiation, axonal growth which end with synaptogenesis and synaptic pruning. Altogether, this leads to the formation of functional neuronal connectivity (Rakic and Lombroso, 1998; Judas et al., 2003, 2005; Kostović and Jovanov-Milošević, 2006; Bystron et al., 2008; Raybaud, 2013; Raybaud et al., 2013). In mouse models, the organization and differentiation of the cortex, neuronal proliferation and migration are essentially complete at birth, and many neurons begin to be eliminated (Bystron et al., 2008; Stiles and Jernigan, 2010; Tau and Peterson, 2010; Raybaud and Widjaja, 2011), influencing the subsequent synaptic connectivity in the brain. Neuronal pruning reaches a peak at two postnatal weeks in mice. In contrast synaptogenesis and neuronal connectivity in the human cortex begin at 22 GW (Semple et al., 2013), but occur mainly after birth, particularly during the first two postnatal years (Huttenlocher, 1979; Herschkowitz et al., 1999). The timing of synaptogenesis in humans is region-dependent, reaching the maximum near postnatal age 3 months in the auditory cortex, 8–12 months in the visual cortex and 2–4 years of age in the prefrontal cortex (Huttenlocher et al., 1982; Lenroot and Giedd, 2006).

The proliferation and migration of neurons shape the coordinated network and connectivity of the developing neocortex. Interneurons migrate from the ganglionic eminences into the cortical plate to form local synapses with cortical pyramidal cells establishing microcircuits (Nadarajah and Parnavelas, 2002). The tangential migration of GABAergic interneurons in the cortex occurs in close association with the radial migration of glutamatergic pyramidal neurons (Bystron et al., 2008). Peak migratory activity of human cortical neurons is suggested to be at mid-gestation (weeks 20–22), though subpopulations of human interneurons continue to migrate through infancy, long after pyramidal neurons have stopped (Bystron et al., 2008; Raybaud et al., 2013). Furthermore, neurogenesis of interneurons occur at later stages of human fetal development (Letinic et al., 2002; Arshad et al., 2016). Late-developing and distinct lineages of GABAergic neurons in the human brain may add to the diversity of inhibitory neuron subtypes and ultimately impact the cortical circuitry that emerges.

The structural and functional development of the cerebral cortex is also regulated by electrical activity (Kirischuk et al., 2017) and connectivity is influenced by early neuronal activity in multiple ways. At embryonic stages, intermittent spontaneous activity is synchronized within small neuronal networks and become more complex during further development of the cerebral cortex, depending on maturation of network connectivity (Egorov and Draguhn, 2013; Yang et al., 2016). Spontaneous synchronous network activity is required to activate silent synapses by incorporating AMPA receptors into the postsynaptic membrane (Durand et al., 1996; Voigt et al., 2005), modeling the functional connectivity within the existing structural network. At this time, the neurotransmitter GABA has an excitatory effect on immature cells and is important in shaping connectivity (Ben-Ari, 2002; Dzhala et al., 2005; Batista-Brito and Fishell, 2009). GABAergic transmission by interneurons contributes to spontaneous network oscillations in the developing cortex through the synapse-driven coordinated activity patterns. In addition, neuronal spontaneous activity regulates GABA synthesis, affecting the inhibitory innervation patterns and the pruning process of redundant neuronal connections (Hata and Stryker, 1994; Chattopadhyaya et al., 2007; Kirischuk et al., 2017).

The connectivity process follows the radial gradient of the inside-out migration of cortical neurons from the deeper to the superficial cortical layers. By 18 GW, when the cortex is still smooth, radial (inside-out), but not tangential (horizontal), intracortical connections have formed (Noctor et al., 2001; Hadders-Algra, 2018). New connections subsequently induce an excessive tangential expansion of the superficial cortical layers (Huttenlocher and Dabholkar, 1997) which is associated with an increase in cortical compressive stress and initiation of cortical folding (Richman et al., 1975). The excessive tangential growth induced by the formation of intracortical horizontal connections is limited to the superficial cortical layers I to IV. As the deep layers and the white matter do not undergo tangential expansion, this process induces compressive stress, which has been hypothesized to lead to surface involution (Tallinen et al., 2014). The late migration of superficial neurons, the increased number of astrocytes, oligodendrocytes, and microglial cells, the intense neural connectivity formation and the laminar organization all contribute to cortical expansion and cortical folding. This process begins around 23 GW following the same tangential gradient as proliferation and connectivity (Huttenlocher et al., 1982; Moulton and Goriely, 2011; Raybaud et al., 2013; Budday et al., 2015b). Therefore, the connectivity-driven tangential growth, as a physics-based approach, mainly affects the superficial layers and induces the formation of gyri and sulci (Huttenlocher, 1979; Raybaud et al., 2013). Through term, secondary gyri formation extend concentrically around the primary sulci and after term, tertiary sulci develop together with short association fibers.

From 22 GW to the end of gestation, the connectivity and circuit organization in the human cortex continue to develop (Marin-Padilla, 1971). The synaptic density increases rapidly after birth, mainly within the early postnatal months and by 2 years of age reaches a level of about 50% higher than that is seen in adulthood (Herschkowitz et al., 1999). Synaptic elimination and remodeling in humans continue to adolescence, while in mice, the entire process appears to be completed by 3–4 weeks of age (Petanjek et al., 2011; Semple et al., 2013). Therefore, the human cortex remains relatively plastic and locally adapts its thickness and stress state as new neuronal connections form and dissolve thought the entire life (Budday et al., 2015a). For example, learning induces the formation of new connections, increases gray matter volume, and changes the brain surface morphology (White et al., 2010), while aging acts in the opposite way.

Neuronal connectivity in the cortex proceeds from the deeper to the superficial cortical layers. Thus, corticothalamic fibers from the deeper layer 6 and corticospinal from layer 5 first project single axons; thalamocortical fibers reach layer 4 and then neurons from layer 3 and 2 form long-association and commissural tracts, receiving multiple incoming fibers (Raybaud et al., 2013). Short-association tracts in the cortical gray matter and in the subcortical white matter results in a horizontal layering pattern of the neocortex (Marin-Padilla, 1970). The developing cortex contains the subplate neurons, the earliest generated neurons in the cerebral cortex of mammals (Luskin and Shatz, 1985; Perkins et al., 2008; Kanold, 2009). In humans, the subplate contains up to 50% of cortical neurons in the second trimester and it remains highly expanded during the first few years of life. The subplate neurons form one of the first functional cortical circuits (Kanold, 2009). They expand markedly during gestation, reaching peak numbers at about gestational week 28, guide efferent axons and establish transient connections with them until their cortical target cells are mature enough to become connected (Shu et al., 2003; Bi et al., 2018; Zhao et al., 2019). As these thalamocortical, corticocortical, interhemispheric commissural fibers and the intracortical connections gradually develop from 26 to 47 weeks, the migration path of the cortical cells traverses the white matter (Raybaud and Widjaja, 2011). Subplate neurons are thus uniquely positioned to establish the initial transient connections between these neurons (Bystron et al., 2008; Budday et al., 2015b).

The most prominent interhemispheric connective structure in the human brain is the corpus callosum (Luders et al., 2010). It begins to differentiate as a commissural plate around week 8, the axons appear around week 12, and adult callosal morphology is achieved around week 20 (Achiron and Achiron, 2001). The genesis of the corpus callosum, which connects the two cerebral hemispheres, depends on the functional integrity of callosal projection neurons and midline cell populations that produce various molecular cues such as semaphorins, netrins, fibroblast growth factors and slits that guide callosal axons to extend toward and cross the midline (Serafini et al., 1996; Bagri et al., 2002; Huffman et al., 2004; Andrews et al., 2006; Smith et al., 2006; Tole et al., 2006; Molyneaux et al., 2009; Niquille et al., 2009; Chinn et al., 2015). The anatomical midline structures that display a guidance activity for callosal axons include the glial wedge, the indusium griseum glia and the hippocampal commissure. The glial wedge, located in the medial wall of the lateral ventricle and composed of astrocytes (Bignami and Dahl, 1973), repels ipsilateral callosal axons toward the midline (Shu and Richards, 2001) and guides the axons toward contralateral cortex (Shu et al., 2003; Keeble et al., 2006). The indusium griseum glia are dorsal to the developing corpus callosum, express SLIT2 and guide commissural axons toward their site of midline crossing (Shu and Richards, 2001). The hippocampal commissure facilitates caudal callosal development, acting as a scaffold for the caudal corpus callosum (Paul et al., 2007).

### Malformations and Cortical Connectivity

Subtle changes in neuronal layering and altered brain connectivity of specific circuits are a common finding in neurological and neuropsychiatric disorders, such as epilepsy, autism spectrum disorder and schizophrenia. Abnormal connectivity has been demonstrated in humans and in animal models by molecular anatomical and neuroimaging studies (Barkovich et al., 2012; Goodkind et al., 2015; Huang et al., 2016; Guarnieri et al., 2018). Recent functional connectivity studies using neuroimaging in humans have demonstrated long-range connectivity defects in patients with variable degrees of cortical malformations. These include patients with epilepsy associated with gray matter heterotopia (Shafi et al., 2015), polymicrogyria (Sethi et al., 2016), focal cortical dysplasia (Jeong et al., 2014; Hong et al., 2017; Rezayev et al., 2018), and tuberous sclerosis complex (Im et al., 2016), patients with schizophrenia (Adhikari et al., 2019; Zhao et al., 2019), autism spectrum disorders (Cooper et al., 2017; Bi et al., 2018) and ACC (Owen et al., 2013). The impaired neuronal connectivity and synaptic plasticity in these diseases have been associated with a decreased expression/function of neural ECM proteins, such as reelin, that might disrupt the axonal guidance cue gradients (Costa et al., 2004; Abdolmaleky et al., 2005; Dong et al., 2005; Berretta, 2012; Folsom and Fatemi, 2013; Jovanov Milošević et al., 2014). The abnormalities in location of particular neuronal populations, cell cues and inputs or presence of abnormal neurons may affect the subsequent developmental steps that control cortical synaptic connectivity (Easter et al., 1985; Redecker et al., 1998; Jacobs et al., 1999b) leading to aberrant interhemispheric, callosal, corticocortical and corticothalamic connectivity (Humphreys et al., 1991; Jacobs et al., 1999a).

Changes in structural connectivity and gyrus formation can also result in an imperfect tangential growth of the cortex and consequent white matter volume reduction. The white matter may become dysplastic together with the cortex, as a part of the cortical malformation, or secondary to the cortical abnormality, and may also become abnormal as a late result of an epileptogenesis process and/or behavioral abnormalities (Budday et al., 2015b). This distorted connectivity, with decreased volume of white matter in the corresponding portion of the hemisphere and the brainstem, has been described as the basis for the aberrant sulcation with no recognizable pattern seen in polymicrogyria (Raybaud and Widjaja, 2011). Microcephaly resulting from a deficient pool of neurons with a consequent lack of connectivity, is frequently associated with abnormalities of the corpus callosum and the level of reduction in white matter volume is correlated with the severity of the malformation (Raybaud and Widjaja, 2011). ACC is a complex condition in which the corpus callosum is partially or completely absent. ACC may or may not be associated with other MCD and can result from any of the following: defects in cellular proliferation and migration, axonal growth or in the midline structures (Jacobs et al., 1999a; Paul et al., 2007).

The synaptic connectivity in the brain is formed by distinct neuronal population interacting in a complex and yet organized spatiotemporal dynamic network. In a rat model for microgyria for example, the permanent loss of all connections coming via white matter, is partially compensated by an increased intracortical connectivity with no changes in the number of cortical neurons (Jacobs et al., 1999a). Such an area with preserved number of neurons and aberrant synaptic connectivity are the most probable sites to generate seizures (Chauvette et al., 2010; Timofeev et al., 2014; Timofeev, 2000). Brain oscillations emerge from this dynamic interaction between intrinsic cellular and network properties and correlate with distinct behavior state (Buzsáki and Draguhn, 2004). Since the circuits necessary for any abnormal oscillations are present in the neocortex, small shifts in the normal network and intrinsically bursting neurons involved in this circuit reorganization could result in either epileptiform activity or abnormal cortical oscillations that would affect the behavior (Chagnac-Amitai and Connors, 1989; Chagnac-Amitai et al., 1990). In the neocortex, recurrent excitatory connections are enhanced in focal cortical dysplasia and the aberrant synaptic connectivity produces a focal epileptogenic zone capable to generate epileptiform activities independent of connections with the malformation itself (Paz and Huguenard, 2015). Aberrant synaptic connectivity and displacement cortical interneurons are associated with abnormalities of gamma oscillations in patients with autism spectrum disorders (An et al., 2018; Hashemi et al., 2018). The neuronal disorganization and clusters of immature neurons in FCD I contribute to hyperexcitability and in the FCD II, in addition to increased excitation caused by immature neurons and reorganization of neuronal network, cytomegalic neurons (FCD II A) intensify the hyperexcitability and recruitment microcircuits in the cortex (Blümcke et al., 2011; Abdijadid et al., 2015). The presence of balloon cells (FCD IIB), claimed to not be involved in epileptogenesis, could play a role in modifying brain oscillation by interfering with neuronal connectivity. Abnormalities of high frequency oscillations and hyperexcitability were recorded in bottom part of the type II FCD cortical sulcus, independent of the presence of the balloon cells (Hu et al., 2019). Interestingly, in FCD I and II, the subcortical white matter neurons are excessive and are particularly frequent just beneath the depth of a sulcus or the base of a gyrus, the U-fiber layer (O’Halloran et al., 2017). The U-fiber system consists of subcortical arcuate fibers, following gyral contour and within gyral cores, originate from pyramidal neurons of layer 6 of cortex, acting as short- synaptic circuits that connect neighboring cortical regions or microcircuits (plexi) (Sarnat, 2018). The neuronal dispersion in the U-fibers compromises the short-range network connections, increasing the microcircuit (plexi) integration and consequently modifying the structure and function of local network contributing to the epileptogenesis.

Neuronal diversity and functional spatiotemporal dynamic in the network are key points to establish the normal connectivity in the brain. The cerebral cortex of mammals has a large diversity of cells operating in intricate circuits. This cellular diversity form complex circuits formed by synapses in distinct cellular compartment and time for encoding processing storage and sending information. Therefore, brain oscillations and behavior depends on the spatiotemporal dynamics of the network (Klausberger and Somogyi, 2008). Aberrant organization, plasticity of neuronal network recruiting distinct microcircuits of different location at specific time could alter synchronicity, leading to abnormal oscillations and consequent behavior, resulting in epilepsy and neuropsychiatric disorders associated with abnormalities of structural and functional connectivity.

## Glial Populations

Neuronal connectivity involves not only the growth of neuronal dendrites and axons, but also the generation and expansion of astrocytes, oligodendrocytes and microglial cells, the formation of synapses, and the development of the vasculature system (Dulla et al., 2013). By 28 weeks of gestation in humans, when excitatory neuronal migration is mostly complete, the number of astrocytes, oligodendrocytes, and microglial cells increases, and myelination reaches its peak that corresponds to the shift from pre-oligodendrocytes to immature oligodendrocytes that produce myelin and induce white matter growth (Semple et al., 2013; Budday et al., 2015b). The white matter development and axonal outgrowth in mice take place only at postnatal day 1–3 which correspond to 23–32 weeks gestation in humans (Craig et al., 2003). Gray matter changes from a radial to a tangential organization during the third trimester. The degree of interconnection within the white matter, and consequent stiffness, remains low between weeks 22 and 38, until term (Raybaud, 2013; Raybaud et al., 2013), but increases after term, when myelination and the formation of astrocytic branches give rise to a highly connected microstructure. In mice, myelination begins during neurogenesis and occurs over a shorter timescale, peaking at approximately postnatal day 20 compared to adolescence in humans (Wiggins, 1986; Rockland and Defelipe, 2011); the increase in white matter stiffness from earlier myelination could in part explain, why mouse brains are less folded than mammalian brains.

Astrocytes with thousands of processes interact with all cell types of the CNS, and help drive nervous system development and sculpt its activity by guiding the migration of developing axons and neurons. Oligodendrocytes provide structural and metabolic support and axon myelination that facilitate nerve impulse conduction. The dynamic regulation of myelination may regulate the precise timing of information propagation and communication across functional circuits. Microglia control cell proliferation, differentiation and modify synapses. Toward the end of the synaptogenic period, weak and inappropriate synapses are eliminated by astrocytes and microglia, leaving neurons with their adult connectivity. The capillaries and neuro-glial-vascular coupling ensure oxygen and metabolic supply to neurons for proper connectivity development (Barres, 2008; Allen and Lyons, 2018). Thus glial dysfunction could contribute to the pathology of MCD via creating a local proinflammatory environment driven by abnormal gliovascular interaction (Kielbinski et al., 2016).

## Conclusion

As our knowledge of human developmental neuroscience increases, we can better understand the heterogeneity and origins of MCD. Common themes that emerge from human studies, when compared to mouse analyses, are the expansion of unique progenitor populations that contribute to neurogenesis and the protracted developmental timeline over which progenitor proliferation and neuronal migration can occur. This has likely provided the substrate to allow for a larger cortex with complex connections, as in the human brain. But it also creates increased vulnerability for mistakes in the neurodevelopmental process at any point. Rodent models remain the foundation for deciphering the precise mechanistic pathways that are implicated in MCD by genetic studies. But a clearer comprehension of how these pathways diverge in the human brain will require the development of creative new approaches to study human neurobiology. Investigations directly on human cortical specimens are crucial to accurately study MCD. There is a need to improve access to tissue samples for research, both from post-mortem tissue specimens and surgical resections. Toward this goal, our scientific community must have ongoing communication with the public about the importance of human tissue-based research. Developing new models for human brain research, including patient-derived 2D and 3D culture systems, and identifying appropriate gyrencephalic animal models, will be fundamental to understanding the pathology of cortical malformations and establish ways to screen treatment approaches. Human cortical development is a long and intricate process but by the same token, it also has the potential for expanded windows of opportunity that may be utilized for therapeutic purposes. More studies are needed to understand this potential.

## Author Contributions

All authors contributed to the ideas, preparation, and editing of this manuscript.

## Conflict of Interest

The authors declare that the research was conducted in the absence of any commercial or financial relationships that could be construed as a potential conflict of interest.
